# Successful Treatment of HER2 V659E Mutation‐Positive Lung Adenocarcinoma With Trastuzumab Deruxtecan: A Case Report

**DOI:** 10.1111/1759-7714.70100

**Published:** 2025-06-04

**Authors:** Mariko Nishihara, Yuki Shinno, Yoshihiro Masui, Ken Masuda, Yuji Matsumoto, Yusuke Okuma, Tatsuya Yoshida, Yasushi Goto, Hidehito Horinouchi, Noboru Yamamoto, Yuichiro Ohe

**Affiliations:** ^1^ Department of Thoracic Oncology National Cancer Center Hospital Tokyo Japan

**Keywords:** ErbB‐2, HER2 V659E, lung adenocarcinoma, trastuzumab deruxtecan

## Abstract

A 67‐year‐old patient with metastatic lung adenocarcinoma harboring a HER2 V659E mutation received trastuzumab deruxtecan (T‐DXd) as second‐line treatment. The V659E mutation is a rare alteration located in the transmembrane domain of HER2. The antitumor effect observed in this patient was comparable to that reported in a previous phase 2 trial, in which most patients had mutations in the kinase domain. Here, we present the detailed clinical course and discuss the findings from a molecular biological perspective.

## Graphical abstract

Clinical course of the patient. Initial imaging showed a 3 cm nodule in the right middle lobe with right pleural effusion and lymphangitic carcinomatosis. First‐line treatment (carboplatin, pemetrexed, durvalumab, tremelimumab) was administered, followed by 2 cycles of tremelimumab maintenance. Disease progression was observed with recurrence of the primary lesion, pleural effusion, and lymphangitic carcinomatosis. Treatment was switched to trastuzumab deruxtecan (T‐DXd). After 5 cycles, the primary lesion shrank, and pleural effusion and lymphangitic carcinomatosis resolved. After the seventh cycle, pleural effusion recurred, leading to discontinuation of T‐DXd.

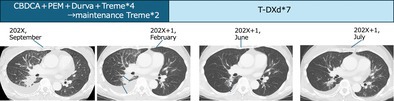

## 1 Introduction

In recent years, the efficacy of trastuzumab deruxtecan (T‐DXd) as a second‐line therapy for human epithelial growth factor receptor type 2 (HER2) mutation‐positive non‐small cell lung cancer (NSCLC) has been demonstrated. HER2 is a receptor tyrosine kinase composed of an extracellular domain, a transmembrane domain (TMD), and a kinase domain [1]. In a clinical trial evaluating the efficacy of T‐DXd in HER2‐mutated NSCLC, most patients (85 of 91, 93%) harbored mutations in the kinase domain and the remaining patients had mutations in the extracellular domain (6 cases, 7%) [2]. In a previous phase 1 pan‐tumor trial, two of 22 NSCLC cases harbored a HER2 G660D mutation, which is located in the TMD; however, no patients in that trial had the V659E mutation [3]. Among the two cases with the G660D mutation, the treatment responses were partial response (PR) and progressive disease (PD) [3]. Therefore, there are currently no data regarding the efficacy of T‐DXd in NSCLC patients harboring a HER2 V659E mutation.

## 2 Case Report

**FIGURE 1:**
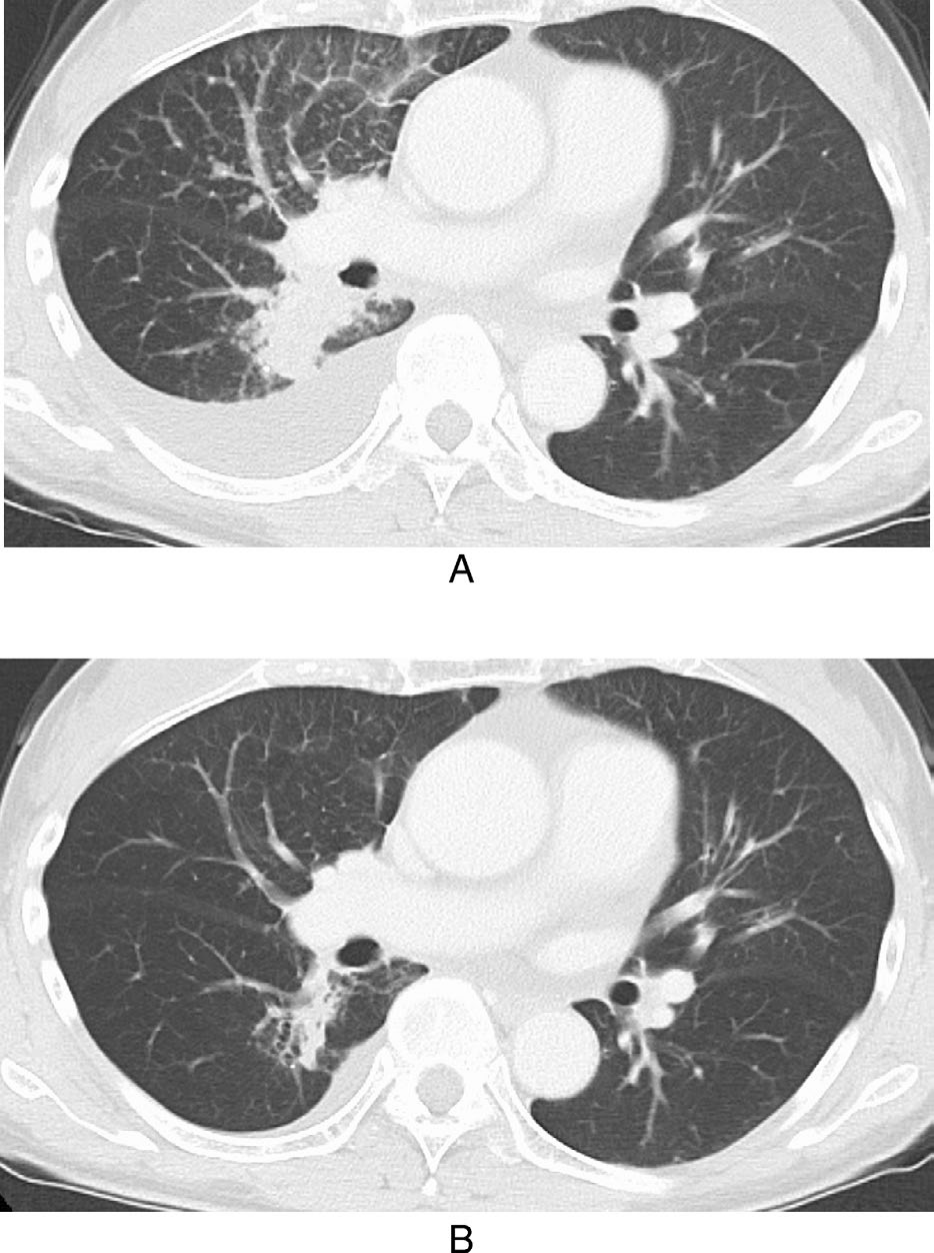
(A) Before initiating T‐DXd, primary lesion, pleural effusion, and lymphangitic carcinomatosis have relapsed, returning to the same findings as before the introduction of first‐line treatment. (B) After 5 cycles of T‐DXd, the primary lesion had shrunk. Pleural effusion and cancerous lymphangitis had almost disappeared.

A 67‐year‐old woman with well‐controlled hypertension and sciatica presented to our hospital with suspected lung cancer. Her performance status was 1. She had a history of smoking two cigarettes per day for 1–2 months 40 years ago. She had no family history of malignancy. Computed tomography (CT) revealed a 3 cm mass in the right middle lobe, mediastinal and hilar lymphadenopathy, and right pleural and pericardial effusions. A transbronchial biopsy led to the diagnosis of cT4N3M1c, clinical stage IVB lung adenocarcinoma. No driver gene mutations were detected using the Oncomine Dx Target Test (ODxTT), and the programmed cell death ligand 1 (PD‐L1) tumor proportion score (TPS) (22C3) was < 1%. The patient was treated with carboplatin, pemetrexed, tremelimumab, and durvalumab as first‐line therapy [4]. The treatment was well tolerated except for grade 1 adrenal insufficiency requiring corticosteroid replacement. After 7 months, CT imaging showed cancerous lymphangitic spread in the right lung, progression of the primary tumor, and recurrence of pleural and pericardial effusions, indicating disease progression (Figure 1A).

Repeat next generation sequencing using the OncoGuide NCC Oncopanel System (Sysmex, Japan) [5] revealed a HER2 V659E mutation. The patient subsequently received T‐DXd at a dose of 5.4 mg/kg every 3 weeks. After 5 cycles, CT demonstrated shrinkage of the primary tumor along with resolution of pleural effusion and lymphangitic carcinomatosis (Figure 1B). The patient achieved a PR, with a 60% reduction in the size of the primary tumor and pleural effusion (Figure 1A,B). However, after 8 cycles of T‐DXd, CT showed new lymphangitic carcinomatosis in the right lower lobe and recurrence of right pleural and pericardial effusions. Progression‐free survival (PFS) was approximately 7 months, and the best response achieved was a PR.

## 3 Discussion

**FIGURE 2:**
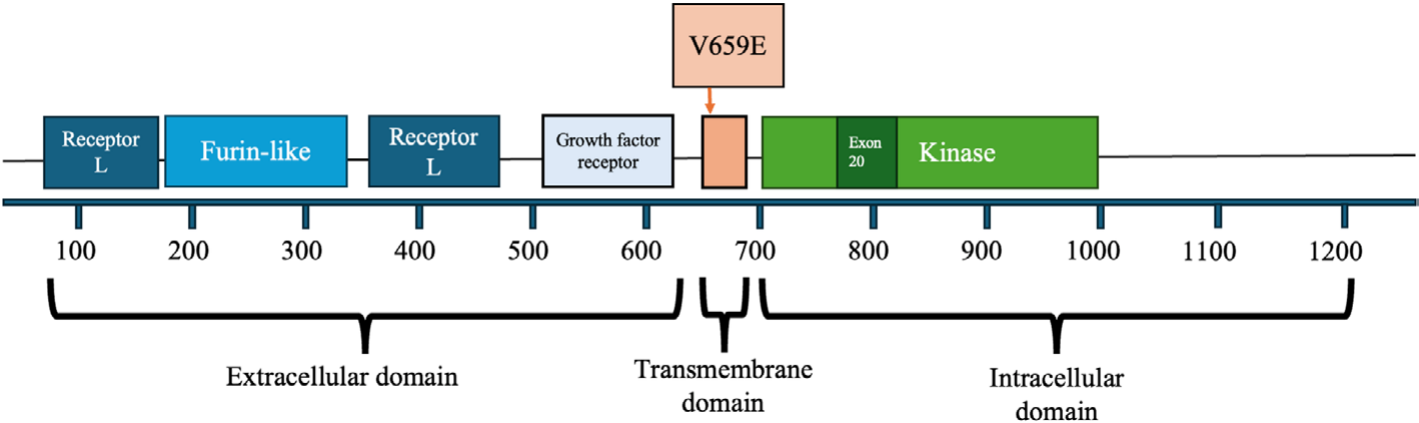
Location of the V659E mutation in the amino acid sequence of the human epidermal growth factor receptor 2 (HER2/ERBB2) [1, 6].

This case involved a patient with lung adenocarcinoma harboring a HER2 V659E mutation located in the TMD (Figure 2). HER2 mutations are found in 1%–4% of lung adenocarcinomas. The most common mutation is an exon 20 insertion in the kinase domain, with a reported frequency of 1%–3% in lung adenocarcinoma [7]. The V659E mutation is detected in 0.09% of lung adenocarcinomas (8 cases out of 8551) [8].

In the pivotal phase 2 clinical trial [2], which is composed of HER kinase domain mutations in over 90% of cases, the objective response rate (ORR) was 55% (95% CI, 44%–65%), with a median PFS of 8.2 months (95% CI, 6.0–11.9 months). Common adverse events included grade 1–2 nausea, fatigue, and alopecia. In another global phase 2 trial evaluating the optimal dose of T‐DXd, the efficacy and safety of two doses, 5.4 and 6.4 mg/kg, were compared. Both doses demonstrated similar efficacy and safety profiles, with nausea, neutropenia, and fatigue being the most common adverse events [9]. In the present case, the PFS was 7 months, with grade 1 nausea and fatigue. Overall, this case demonstrated that T‐DXd at 5.4 mg/kg was effective for the HER2 V659E mutation with an acceptable toxicity profile comparable to those reported in pivotal clinical trials.

HER2 typically forms heterodimers with other ERBB family receptors or homodimers with itself, leading to the activation of downstream tyrosine kinase signaling cascades. An exon 20 insertion mutation alters the position of the αC helix in the kinase domain, resulting in constitutive dimerization and activation of downstream signaling pathways [10]. Mutations in the TMD also promote dimerization and activation of downstream signaling, classifying them as pathogenic. Molecular biology studies have suggested that the V659E mutation induces structural changes that stabilize the dimeric structure and alter the polarity of the N‐terminal site of the TMD, thereby stabilizing dimerization and activating the downstream tyrosine kinase pathway [8, 11]. Several reports have indicated that tyrosine kinase inhibitors are effective in patients with the V659E mutation [12, 13]. Furthermore, the V659E mutation may enhance drug internalization. In preclinical studies comparing the HER2 internalization rate between HER2 wild‐type and HER2 mutation‐positive models, some mutation‐positive models, including those with the G660D mutation located in the TMD, exhibited a two‐fold increase in internalization rate [14]. Additionally, application materials submitted to the Pharmaceuticals and Medical Devices Agency reported that the internalization rate of the V659E mutation is comparable to that of major mutations. These findings suggest that the V659E mutation activates HER2, increases T‐DXd internalization, and contributes to an antitumor effect similar to that observed in cases with previously reported major mutations.

In this case, the HER2 mutation was detected by repeat NGS. Adenocarcinoma in nonsmokers has a higher likelihood of harboring actionable genetic mutations detectable by NGS than adenocarcinoma in smokers [15]. Repeat NGS may, therefore, be beneficial, particularly for nonsmokers with adenocarcinoma, even after an initial negative NGS result.

In conclusion, we report a patient with NSCLC harboring a rare HER2 V659E mutation in the TMD who responded well to T‐DXd, with a safety profile comparable to those observed in cases with common HER2 mutations.

## Author Contributions

Conception and design: Mariko Nishihara, Yuki Shinno. Provision of study material or patients: no. Collection and assembly of data: Mariko Nishihara, Yuki Shinno. Data analysis and interpretation: All authors. Manuscript writing: All authors. Final approval of manuscript: All authors. Accountable for all aspects of the work: All authors.

## Ethics Statement

All procedures involving human participants adhered to the ethical standards of the institutional and/or national research committee and the 1964 Declaration of Helsinki.

## Consent

Written consent was obtained from the patient in this case report to allow the publication of her anonymized details in the literature.

## Conflicts of Interest

Y.S. reports receiving personal fees from Bristol‐Myers Squibb, Chugai, AstraZeneca, and Eli Lilly; grants and personal fees from Ono; and grants from Janssen and Japan Clinical Research Operations K.K. outside of the submitted work. K.M. reports receiving personal fees from Ono Pharmaceutical Co. Ltd., AstraZeneca, Chugai, and Bristol‐Myers Squibb, outside of the submitted work. T.Y. reports receiving grants and personal fees from Amgen, AstraZeneca, Ono, Merck Sharp & Dohme, Novartis, Chugai, and Bristol‐Myers Squibb; grants from Takeda, Daiichi Sankyo, and AbbVie; and personal fees from Taiho, Eli Lilly, Roche, and ArcherDX outside of the submitted work. Y.M. reports receiving grants from the National Cancer Center Research and Development Fund, Grant‐in‐Aid for Scientific Research on Innovative Areas, and Hitachi Ltd.; grants and personal fees from Olympus; and personal fees from AstraZeneca, Novartis, COOK, AMCO Inc., Thermo Fisher Scientific, Erbe Elektromedizin GmbH, Fujifilm, Chugai, and Eli Lilly outside of the submitted work. Y.O. reports receiving grants from Roche and AbbVie K.K.; and personal fees from AstraZeneca, Ely Lilly K.K., Bristol‐Myers Squibb, Pfizer Taiho Pharma Co. Ltd., AstraZeneca Nippon Boehringer Ingelheim, Chugai Pharma Co. Ltd., Ono Pharma Co. Ltd., and Taiho Pharma Co. Ltd. outside of the submitted work. Y.G. reports receiving grants from AZK, AbbVie, Kyorin, and Preferred Network; grants and personal fees from Pfizer, Eli Lilly, Bristol‐Myers Squibb, Ono, Novartis, and Daiichi Sankyo; and personal fees from Chugai, Taiho, Boehringer Ingelheim, Merck Sharp & Dohme, Merck, Thermo Fisher, AstraZeneca, Chugai, Guardant Health Inc., and Illumina outside of the submitted work. H.H. reports receiving grants and personal fees from Merck Sharp & Dohme, AstraZeneca, Ono, Chugai, Roche, and Novartis; grants from AbbVie, Bristol‐Myers Squibb, Merck Biopharma, Daiichi Sankyo, Janssen, and Genomic Health; and personal fees from Eli Lilly and Kyowa‐Kirin, outside of the submitted work. N.Y. reports receiving grants from Chugai, Taiho, Eisai, Eli Lilly, Quintiles, Astellas, Bristol‐Myers Squibb, Novartis, Daiichi Sankyo, Pfizer, Boehringer Ingelheim, Kyowa‐Hakko Kirin, Bayer, Ono Pharmaceutical Co. Ltd., Takeda, Janssen Pharma, Merck Sharp & Dohme, Merck, GlaxoSmithKline, Sumitomo Dainippon, Chiome Bioscience Inc., Otsuka, Carna Biosciences, Genmab, and Shionogi; and personal fees from Ono Pharmaceutical Co. Ltd., Chugai, AstraZeneca, Pfizer, Lilly, Bristol‐Myers Squibb, Eisai, Otsuka, Takeda, Boehringer Ingelheim, Cimic, Sysmex, and Eisai, outside of the submitted work. Y.O. reports receiving grants, personal fees, and nonfinancial support from AstraZeneca, Chugai, Ono Pharmaceutical Co. Ltd., and Bristol‐Myers Squibb; grants and personal fees from Eli Lilly and Pfizer; grants and nonfinancial support from Kyorin; grants from Dainippon‐Sumitomo, Taiho, Novartis, Takeda, Kissei, Daiichi Sankyo, Janssen, and LOXO; and personal fees from Boehringer Ingelheim, Bayer, Merck Sharp & Dohme, Taiho, Nippon Kayaku, Kyowa‐Hakko Kirin, Celltrion, Amgen, and AnHeeart Therapeutics Inc. outside of the submitted work. The other authors declare no conflicts of interest.

## Data Availability

Data available on request due to privacy/ethical restrictions.
